# Endodontic Surgery of a Symptomatic Overfilled MTA Apical Plug: A Histological and Clinical Case Report

**DOI:** 10.22037/iej.v12i3.17689

**Published:** 2017

**Authors:** Saeed Asgary, Sara Fayazi

**Affiliations:** a *Iranian Center For Endodontic Research, Research Institute of Dental Sciences, Dental School, Shahid Beheshti University of Medical Sciences, Tehran, Iran; *; b *The University of Texas Health Science Center at San Antonio, Texas, USA*

**Keywords:** Apical barrier, Apicoectomy, Calcium-Enriched Mixture, CEM Cement, Endodontics, MTA, Surgical treatment

## Abstract

This case report presents the successful surgical treatment of a symptomatic open apex upper central incisor with a failed overfilled mineral trioxide aggregate (MTA) apical plug. Unintentional overextension of the MTA had occurred two years before the initial visit. An apical lesion adjacent to the excess MTA was radiographically detectable. Endodontic surgery was performed using calcium-enriched mixture (CEM) cement as a root-end filling material. Curettage of the apical lesion showed a mass of unset MTA particles; histopathological examination revealed fragments of MTA and granulation tissues. Up to 18-month follow-up, the tooth was clinically asymptomatic and fully functional. Periapical radiograph and CBCT images showed a normal periodontal ligament around the root. In conclusion, favorable outcomes in this case study suggested that root-end filling with CEM cement might be an appropriate approach; in addition, however many factors probably related to the initial failure of the case, the extrusion of MTA into the periapical area should be avoided.

## Introduction

Traditionally, calcium hydroxide (CH) has been the material of choice for the management of non-vital teeth with open apices. However, CH apexification has several disadvantages: it takes several appointments to replace CH, there are concerns about patient cooperation, evidence shows dentin brittleness and coronal microleakage when CH is used, and there is an increased risk of cervical root fracture [1]. Due to its shortcomings, CH has been somewhat replaced with novel biomaterials during the last decade [2, 3]. An alternative treatment to multi-session CH therapy is a one-visit apexification approach, which is completed by inserting an artificial barrier material in the apical portion of an open apex tooth. Currently, mineral trioxide aggregate (MTA) is considered the biomaterial of choice due to its sealability, biocompatibility and hard-tissue inductivity with favorable treatment outcomes [4, 5].

Obtaining an optimal apical seal in teeth with immature apices is challenging due to the wide apical foramen that requires a large volume of filling material that may extrude into the periradicular tissue thus eliciting foreign-body reactions [6]. Special placement techniques, using manual, ultrasonic or ultrasonic-assisted hand delivery for MTA have been suggested to minimize extrusion of the material [7, 8]. Although favorable clinical outcomes from one-visit MTA apexification have been reported [9-11], a few case reports have shown that endodontic lesions will not heal when the filling materials overextend the root canal during orthograde obturation [12, 13]. An animal histologic study by Torabinejad *et al.* [14] showed that MTA implanted into the animal bone resulted in minimal inflammatory reactions with favorable bone healing with direct bone apposition. On the other hand, Holland *et al.* [6] described that over extrusion of the MTA into the periapical tissue decrease the chance of success of endodontic treatment. Calcium-enriched mixture (CEM) cement is water-based and tooth-colored endodontic biomaterial. It is biocompatible, sets in the presence of moisture and blood and provides a good seal. When used as an artificial barrier material for one-visit apexification, CEM has shown promising outcomes; in addition, as a root-end filling or perforation repair material, it stimulates cementogenesis and osteogenesis [15, 16].

**Figure 1.**
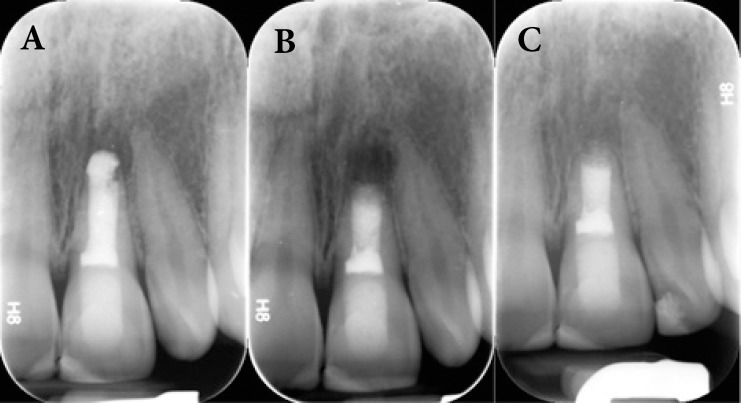
A) Preoperative periapical radiograph of endodontically treated right central incisor; B) Periapical radiograph after CEM cement root-end filling; C) Periapical radiograph after one year follow-up shows complete bone healing and establishment of periodontal ligament (PDL

This case report describes the successful surgical treatment of a symptomatic immature upper central incisor tooth with an overextended MTA apical plug and a periapical lesion using CEM cement.

## Case Report

**Figure 2:**
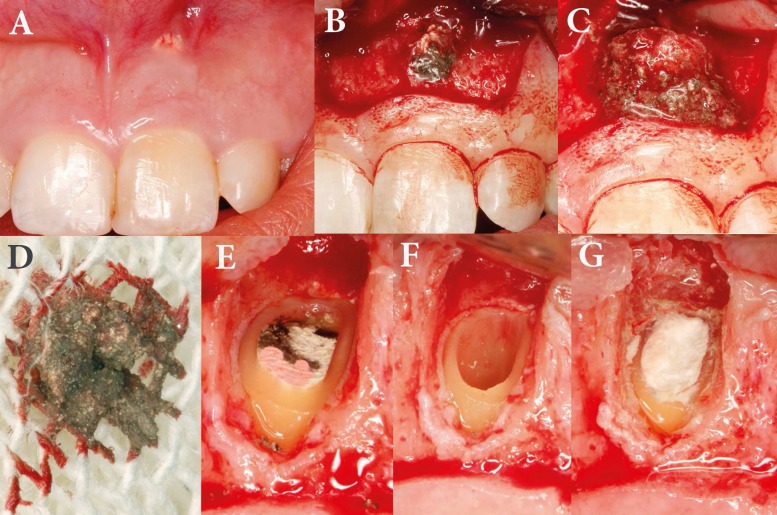
A) Preoperative clinical view of treated right central incisor; B and C) Clinical view of the unset MTA overextrusion and granulation tissue after flap reflection; D) Removed mass of MTA overextrusion; E) Resected apex; F) Preparation of the root-end cavity and removing the previous root canal fillings; G) Placement of CEM cement as the root-end filling biomaterial

A 24-year-old female with a noncontributory medical history was referred to the Mehr Dental Clinic due to periodic swelling and discomfort associated with her upper left central incisor. The report from the referring dentist noted that the tooth had been treated with a white ProRoot MTA plug (Dentsply Tulsa Dental, Tulsa, OK) two years ago. The records detailed a history of impact trauma and pulp necrosis 17 years ago. Initial radiographs showed an immature tooth with a periapical rarefaction and an extruded MTA plug (Figure 1A). Intraoral examination revealed sensitivity to palpation of the tissues adjacent to the apex of the tooth. The periodontium was normal with no mobility. Slight tooth discoloration was evident (Figure 2A). According to the clinical and radiographic findings, the final clinical diagnosis was symptomatic apical periodontitis with a failed overfilled MTA apical plug.

The patient was presented with several treatment options including tooth extraction and implant replacement. However, patient elected to save the tooth, and therefore, endodontic surgery was recommended. The risk and benefits of the proposed treatment were described, and informed consent was obtained.

**Figure 3.**
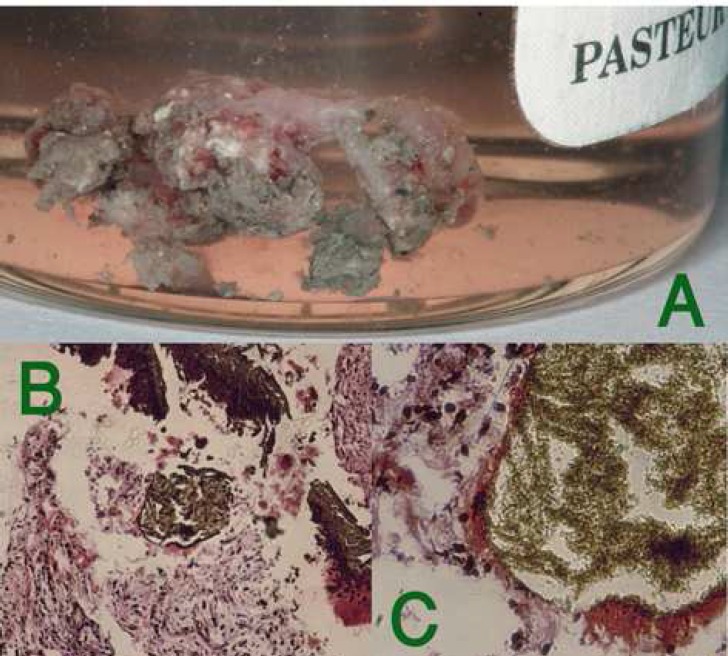
) Mass of unset MTA extruded attached to the soft tissue removed from periapical tissue; B) MTA surrounded by granulation tissue, macrophage and fibroblasts; C) MTA particles encapsulated in the fibrous connective tissue

The surgical procedure was performed by an Endodontist (S.A.). The patient was anesthetized with 2% lidocaine with 1:80,000 epinephrine (DarouPakhsh, Tehran, Iran) and an Ochsenbein Luebke flap was raised. Following reflection of the flap and initial curettage, a mass of dark unset MTA was found (Figure 2B to D). MTA remnants and granulation tissue were completely removed, stored in formalin solution and submitted for pathological examination (Figure 3A). After root-end resection (~1 mm), four different zones were identified in the root canal space: a washed out area of empty space, the set MTA filling, the unset MTA filling and the pink gutta-percha filling (Figure 2E). The root-end was prepared with an ultrasonic retrotip (Joya electronics, Tehran, Iran), (Figure 2F). CEM cement (BioniqueDent, Tehran, Iran) powder and liquid were mixed according to the manufacturer’s instruct-tions; the cement was delivered into the root-end cavity using a plastic instrument (Figure 2G). After root-end sealing and radiographic confirmation of the proper root-end filling (Figure 1B), the flap was repositioned and sutured. Five days later, the patient returned symptom free. Histo-pathological assessment verified granulation tissue with fragments of MTA particles, which were encapsulated within fibrous connective tissue (Figure 3B and C).

**Figure 4:**
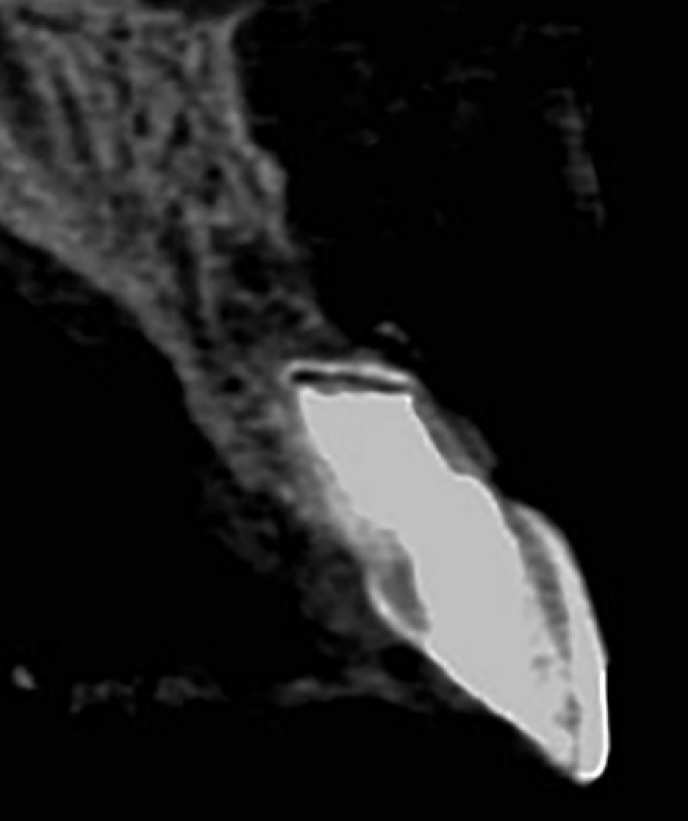
Cone-beam computed tomography (CBCT) image shows the complete healing of periapical lesion and PDL reformation 18 months after root-end filling with CEM cement

The patient presented for a recall appointment 1 year later. Clinical examination revealed no sensitivity to percussion or palpation, and the tooth was fully functional with normal probing depths. Radiographic examination showed the apical lesion had healed with a normal PDL established (Figure 1C). The CBCT image at the 18 month follow-up evaluation revealed an excellent treatment outcome with regeneration of the periradicular tissue (Figure 4). 

## Discussion

The presented case shows successful surgical management of a symptomatic central incisor with an open apex, poorly sealed apical filling and overextended unset MTA apical plug. In this case a considerable amount of MTA was extruded into the apical area during placement. MTA might be dislodged through the wide apical foramen or be pushed actively beyond the apical foramen. The extruded MTA seems to have been compressed or moved out from its original location. 

In some case reports with at least 12 months follow-up, apexification and periodical healing occurred even when a considerable amount of MTA was extruded into the periodical tissue [17, 18]. Sealing the blunderbuss canals is the key factor in the successful treatment of immature necrotic teeth. However, adequate compaction of MTA in such teeth is challenging. In the present case, the orthograde approach resulted in an inadequate seal of the canal. The modified internal matrix concept is likely to facilitate compaction of repair material and prevent extrusion of the material into periapical tissues [19-21]; it also prevents blood-contamination of MTA and allows proper compaction of the material using resorbable mineral-organic biomaterials. Calcium sulfate and Collaplug have been used as such an internal matrix [21]. Also the short-term application of Ca(OH)2 prior to apexification might have additional benefits but has shown to be strongly related to the extrusion of MTA and formation of barriers beyond the limits of the root canal walls [22].

Revitalization and retreatment were not attempted for the present case because complete removal of root canal filling materials (ProRoot MTA, gutta-percha, and endodontic sealer) from root canals and periradicular tissue without causing damage was impossible. Furthermore, previous root canal preparation, and particularly, overfilled unset MTA might have had adverse effects on stem cells and periapical healing. In addition, current literature regarding regenerative endodontics in retreatment cases is scarce.

The nature of periradicular tissue fluids (*i.e.* blood, serum and pus) as well as the pH of the treatment anatomy can affect the chemo-physical properties of the MTA [23-26]. Researchers have found that acidic solutions have a destructive effect on the physico-mechanical properties of MTA. Therefore, the sealing ability, push-out bond strength and surface hardness of MTA decrease significantly in the acidic environments [26-29].

In addition, blood contamination negatively influences the formation of CH in the early stage of MTA hydration process [24]. CH interacts with PO4- originated from tissue fluids and produces amorphous calcium phosphate precipitation, which finally induces hydroxyapatite crystal formation [28]. These byproducts fill the MTA-dentin interface and create a biologic seal in addition to mechanical seal. In the present case, it seems the sealing ability of MTA decreased significantly in direct contact with blood and pus (acidic pH) [29]. Therefore, the main objective of one-visit apexification is not met with one-visit MTA application. Furthermore, it is supposed that extruded MTA may stay unset [18]. The tissue response to unset MTA particles is probably different from that of a biocompatible set material. So, extruded unset MTA may be associated with ongoing periapical irritation. In this case, the histopathological report revealed that MTA particles were encapsulated within fibrous connective tissue which might act as a physical irritant to the tissue. The outcome of treatment after extrusion of MTA into the periradicular tissues is unpredictable, and more clinical studies on tissue reactions to unset MTA are recommended. 

Calcium-enriched mixture cement has been recently introduced as a hydrophilic tooth colored endodontic material. CEM cement is composed of calcium oxide, calcium sulfite, phosphorus oxide and silica as major elements [30]. It has been shown that CEM cement is an alkaline biomaterial with superiorantibacterial properties compared to MTA [31], which releases CH during and after setting [32]. It has been shown that the sealing ability of CEM cement and MTA as root-end filling materials is similar [30]. 

This paper describes the treatment outcomes of CEM cement as a root-end filling material in a failed apexification. In the presented case, use of CEM cement as a root-end filling material showed successful clinical and radiographic outcomes with 18 month follow-up. The CBCT image shows the complete healing of apical lesion and reformation of the PDL. This is consistent with the studies have shown that, like MTA, PDL regeneration, osteogenesis, cementogenesis and dentino-genesis occur in contact with CEM cement [30, 33-35].

## Conclusion

In conclusion, there is a chance that overextrusion of MTA can cause delay in healing of the apical lesion, and MTA can left unset in the periapical lesion. Regarding the advantages of CEM cement over MTA including improved color, easier handling, lower cost and antibacterial effects, CEM may be considered as a proper biomaterial to be used as a root-end filling biomaterial in teeth with open apices. However, more clinical studies with longer follow-ups are recommended.

## References

[1] Rafter M (2005). Apexification: a review. Dent Traumatol.

[2] El-Meligy OA, Avery DR (2006). Comparison of apexification with mineral trioxide aggregate and calcium hydroxide. Pediatr Dent.

[3] Witherspoon DE, Ham K (2001). One-visit apexification: technique for inducing root-end barrier formation in apical closures. Pract Proced Aesthet Dent.

[4] (2009). Evidenced-based review of clinical studies on the root apex. J Endod.

[5] Damle SG, Bhattal H, Loomba A (2012). Apexification of anterior teeth: a comparative evaluation of mineral trioxide aggregate and calcium hydroxide paste. J Clin Pediatr Dent.

[6] Holland R, Mazuqueli L, de Souza V, Murata SS, Dezan Junior E, Suzuki P (2007). Influence of the type of vehicle and limit of obturation on apical and periapical tissue response in dogs' teeth after root canal filling with mineral trioxide aggregate. J Endod.

[7] Bogen G, Kuttler S (2009). Mineral trioxide aggregate obturation: a review and case series. J Endod.

[8] Yeung P, Liewehr FR, Moon PC (2006). A quantitative comparison of the fill density of MTA produced by two placement techniques. J Endod.

[9] Giuliani V, Baccetti T, Pace R, Pagavino G (2002). The use of MTA in teeth with necrotic pulps and open apices. Dent Traumatol.

[10] Maroto M, Barberia E, Planells P, Vera V (2003). Treatment of a non-vital immature incisor with mineral trioxide aggregate (MTA). Dent Traumatol.

[11] D'Arcangelo C, D'Amario M (2007). Use of MTA for orthograde obturation of nonvital teeth with open apices: report of two cases. Oral Surg Oral Med Oral Pathol Oral Radiol Endod.

[12] Brito-Junior M, Faria-e-Silva AL, Quintino AC, Moreira-Junior G, Geber M, Camilo CC, Soares JA (2012). Orthograde retreatment failure with extruded MTA apical plug in a large periradicular lesion followed by surgical intervention: case report. Gen Dent.

[13] Asgary S, Eghbal MJ, Mehrdad L, Kheirieh S, Nosrat A (2014). Surgical management of a failed internal root resorption treatment: a histological and clinical report. Restor Dent Endod.

[14] Torabinejad M, Ford TR, Abedi HR, Kariyawasam SP, Tang HM (1998). Tissue reaction to implanted root-end filling materials in the tibia and mandible of guinea pigs. J Endod.

[15] Nosrat A, Asgary S, Eghbal MJ, Ghoddusi J, Bayat-Movahed S (2011). Calcium-enriched mixture cement as artificial apical barrier: A case series. J Conserv Dent.

[16] Tabrizizade M, Asadi Y, Sooratgar A, Moradi S, Sooratgar H, Ayatollahi F (2014). Sealing ability of mineral trioxide aggregate and calcium-enriched mixture cement as apical barriers with different obturation techniques. Iran Endod J.

[17] Tahan E, Celik D, Er K, Tasdemir T (2010). Effect of unintentionally extruded mineral trioxide aggregate in treatment of tooth with periradicular lesion: a case report. J Endod.

[18] Nosrat A, Nekoofar MH, Bolhari B, Dummer PM (2012). Unintentional extrusion of mineral trioxide aggregate: a report of three cases. Int Endod J.

[19] Lemon RR (1992). Nonsurgical repair of perforation defects Internal matrix concept. Dent Clin North Am.

[20] Bargholz C (2005). Perforation repair with mineral trioxide aggregate: a modified matrix concept. Int Endod J.

[21] Al-Daafas A, Al-Nazhan S (2007). Histological evaluation of contaminated furcal perforation in dogs' teeth repaired by MTA with or without internal matrix. Oral Surg Oral Med Oral Pathol Oral Radiol Endod.

[22] Felippe WT, Felippe MC, Rocha MJ (2006). The effect of mineral trioxide aggregate on the apexification and periapical healing of teeth with incomplete root formation. Int Endod J.

[23] Namazikhah MS, Nekoofar MH, Sheykhrezae MS, Salariyeh S, Hayes SJ, Bryant ST, Mohammadi MM, Dummer PM (2008). The effect of pH on surface hardness and microstructure of mineral trioxide aggregate. Int Endod J.

[24] Nekoofar MH, Davies TE, Stone D, Basturk FB, Dummer PM (2011). Microstructure and chemical analysis of blood-contaminated mineral trioxide aggregate. Int Endod J.

[25] Mirhadi H, Moazzami F, Safarzade S (2014). The Effect of Acidic pH on Microleakage of Mineral Trioxide Aggregate and Calcium-Enriched Mixture Apical Plugs. Iran Endod J.

[26] Mohebbi P, Asgary S (2016). Effect of pH on physical properties of two endodontic biomaterials. J Conserv Dent.

[27] Shokouhinejad N, Nekoofar MH, Iravani A, Kharrazifard MJ, Dummer PM (2010). Effect of acidic environment on the push-out bond strength of mineral trioxide aggregate. J Endod.

[28] Sarkar NK, Caicedo R, Ritwik P, Moiseyeva R, Kawashima I (2005). Physicochemical basis of the biologic properties of mineral trioxide aggregate. J Endod.

[29] Saghiri MA, Lotfi M, Saghiri AM, Vosoughhosseini S, Fatemi A, Shiezadeh V, Ranjkesh B (2008). Effect of pH on sealing ability of white mineral trioxide aggregate as a root-end filling material. J Endod.

[30] Asgary S, Ahmadyar M (2013). Vital pulp therapy using calcium-enriched mixture: An evidence-based review. J Conserv Dent.

[31] Asgary S, Kamrani FA (2008). Antibacterial effects of five different root canal sealing materials. J Oral Sci.

[32] Asgary S, Eghbal MJ, Parirokh M, Ghoddusi J (2009). Effect of two storage solutions on surface topography of two root-end fillings. Aust Endod J.

[33] Rahimi S, Mokhtari H, Shahi S, Kazemi A, Asgary S, Eghbal MJ, Mesgariabbasi M, Mohajeri D (2012). Osseous reaction to implantation of two endodontic cements: Mineral trioxide aggregate (MTA) and calcium enriched mixture (CEM). Med Oral Patol Oral Cir Bucal.

[34] Mehrdad L, Malekafzali B, Shekarchi F, Safi Y, Asgary S (2013). Histological and CBCT evaluation of a pulpotomised primary molar using calcium enriched mixture cement. Eur Arch Paediatr Dent.

[35] Asgary S, Ehsani S (2013). Periradicular surgery of human permanent teeth with calcium-enriched mixture cement. Iran Endod J.

